# Curdlan-Reinforced Chitosan/Polyacrylate Interpenetrating Hydrogels with Enhanced Mechanical Stability for Gastric Retention and pH-Responsive Drug Release

**DOI:** 10.3390/gels12050378

**Published:** 2026-04-30

**Authors:** Yuzhong Feng, Peng Wu, Ping Zhang, Ni Wang, Ke Wang, Shuye Qi, Xiaodong Chen

**Affiliations:** 1 Life Quality Engineering Interest Group, School of Chemical and Environmental Engineering, College of Chemistry, Chemical Engineering and Materials Science, Soochow University, Suzhou 215123, China; 20235209003@stu.suda.edu.cn (Y.F.); xdchen@suda.edu.cn (X.C.); 2 Shenzhen X-Institute, Shenzhen 518055, China; 3 National Institutes for Food and Drug Control, Beijing 102629, China; 4 Institute of Biopharmaceutical and Health Engineering (iBHE), Tsinghua Shenzhen International Graduate School, Tsinghua University, Shenzhen 518055, China

**Keywords:** chitosan-based hydrogel, curdlan, metformin hydrochloride, controlled release, gastric retention, swelling capacity

## Abstract

Polysaccharide-based hydrogels for gastric retention face the inherent challenge of achieving effective retention through swelling while avoiding mechanical failure. Here, we introduce a strategy by incorporating curdlan into chitosan/sodium polyacrylate interpenetrating networks to reinforce the hydrogel and regulate swelling-induced transport behavior. Curdlan-reinforced chitosan/polyacrylate (CS/CUR/PAAS) hydrogels with varying curdlan content (0–4 wt.%) were synthesized and characterized. Optimal reinforcement was achieved with 2 wt.% curdlan, yielding an indentation hardness of ~80 kPa and an elastic modulus of ~63 kPa without compromising swelling capacity. Under acidic conditions (pH 1.2), the hydrogel swelled rapidly (~50-fold at 3 h; ~140-fold at 8 h) while maintaining structural integrity. Using a dynamic in vitro human stomach simulator (DHSI-IV), the optimized hydrogel demonstrated gastric retention for up to 5 h, with ~60% of the initial mass retained at 6 h. Metformin hydrochloride release followed diffusion-controlled kinetics (~69% over 8 h), governed primarily by pH with secondary shear modulation. Microstructural and rheological analyses revealed that acidic conditions regulated network expansion, viscoelastic relaxation, and pore formation, which in turn controlled transport pathways and drug release. The findings highlight that curdlan reinforcement stabilizes swelling behavior under acidic conditions, offering a robust and pH-responsive strategy for designing mechanically stable, gastric-retentive hydrogels.

## 1. Introduction

Hydrogels constructed from natural polysaccharides have attracted extensive attention as functional soft materials because of their biocompatibility, structural diversity, and capacity to form responsive three-dimensional networks in aqueous environments [1,2]. The physicochemical properties of such hydrogels, including swelling behavior, mechanical stability, and molecular transport, are strongly governed by the structure and interactions of the constituent polysaccharide chains. Understanding how polysaccharide architecture and network organization regulate these properties is therefore essential for the rational design of hydrogel systems for biomedical and food-related applications.

Acid-responsive, swellable hydrogels have been widely investigated for gastric-retentive drug delivery. Under acidic conditions, protonation of ionizable groups induces polymer chain expansion and water uptake, increasing hydrogel volume and prolonging gastric residence [3,4]. However, excessive swelling often leads to mechanical weakening or erosion under the compressive and shear stresses generated by gastric motility. Achieving a balance between swelling capacity and mechanical stability therefore remains a major challenge, particularly for highly water-soluble drugs such as metformin hydrochloride, which undergo rapid dissolution and burst release in acidic media [5,6]. Although sustained-release formulations exist [7], maintaining predictable release in mechanically dynamic gastric environments remains difficult when hydrogel matrices undergo excessive swelling or structural relaxation [8].

Chitosan is a linear natural cationic polysaccharide composed of D-glucosamine and N-acetyl-D-glucosamine units linked by β-(1,4)-glycosidic bonds. The degree of deacetylation, as its core structural parameter, directly determines the content of free amino groups on the molecular chain, which serves as the fundamental structural basis for the ionic crosslinking ability, pH responsiveness, biocompatibility, and mucoadhesive properties of chitosan [9]. Owing to its pH-dependent solubility and excellent mucoadhesive features, chitosan has been extensively investigated for the development of gastric retention drug delivery systems [10,11]. Under acidic conditions, protonation of amino groups induces polymer chain expansion and water uptake, thereby facilitating swelling and gastric retention [12]. Nevertheless, chitosan-based hydrogels often exhibit limited mechanical strength and insufficient resistance to prolonged acidic exposure, resulting in network relaxation or uncontrolled drug release [13]. Structural reinforcement strategies are therefore required to enhance mechanical stability while maintaining acid responsiveness.

Metformin hydrochloride (MET) is a first-line oral therapeutic agent for type 2 diabetes mellitus with a short elimination half-life (1.5–4.5 h), requiring frequent dosing and leading to poor patient compliance. In addition, its rapid gastric dissolution and narrow absorption window make it highly sensitive to carrier structure and an ideal probe for evaluating gastric-retentive and diffusion-regulated release systems [14]. Therefore, MET serves as a representative model to assess whether the designed hydrogel system can simultaneously achieve prolonged gastric retention and controlled release under acidic conditions.

Composite hydrogel approaches incorporating secondary polysaccharides offer a promising solution for improving gastric-retentive performance [15,16]. Curdlan (CUR), a linear β-1,3-glucan produced by microbial fermentation, undergoes irreversible thermal gelation to form a rigid triple-helical network with high stiffness and elastic recovery [17,18]. Specifically, upon heating above 80 °C, CUR assembles into a highly ordered β-(1 → 3) triple-helix structure that remains stable and resistant to hydrolysis under strongly acidic gastric conditions. Compared with other β-glucans (e.g., yeast-derived β-(1 → 3)/(1 → 6)-glucan), CUR exhibits a higher degree of chain ordering and superior gel strength, which translates into enhanced structural stability under mechanical and acidic stress [17]. In contrast to synthetic crosslinkers such as glutaraldehyde, CUR is a food-grade, FDA-approved biopolymer that does not require covalent chemistry or reactive coupling agents, thereby avoiding cytotoxicity concerns and irreversible over-crosslinking. Importantly, CUR forms a physically crosslinked interpenetrating polymer network with chitosan and sodium polyacrylate (PAAS) through hydrogen bonding and chain entanglement, resulting in simultaneous enhancement of storage modulus and resistance to deformation under acidic conditions [19,20]. Within this system, CUR acts as a reinforcing skeletal phase that stabilizes the hydrogel network while preserving biocompatibility. In addition, PAAS functions as a highly swellable polyelectrolyte that drives water uptake through osmotic pressure and electrostatic repulsion, thereby expanding network porosity and facilitating mass transport [21]. The combination of chitosan, CUR, and PAAS therefore establishes a tunable interpenetrating platform in which swelling behavior, mechanical reinforcement, and diffusion pathways can be independently regulated for gastric-retentive applications [17,22].

Despite these advantages, how reinforcing components regulate the coupling between swelling, mechanical stability, and molecular transport under physiologically relevant gastric conditions remains insufficiently understood. Most studies rely on static measurements that inadequately capture the combined effects of gastric acidity, peristaltic shear, and dynamic emptying on hydrogel behavior [23,24,25]. Consequently, the structure-property-performance relationships governing gastric retention and controlled release in mechanically dynamic environments are not yet well established.

In this study, we developed a curdlan-reinforced chitosan/sodium polyacrylate (CS/CUR/PAAS) composite hydrogel as a gastric-retentive delivery system, using metformin hydrochloride as a model highly water-soluble drug. We hypothesized that controlled curdlan incorporation would establish a mechanically reinforced yet acid-responsive network that stabilizes hydrogel structure during swelling and regulates diffusion pathways. Through systematically varying curdlan concentration, we elucidated relationships among network microstructure, swelling, mechanical properties, gastric retention, and release kinetics. Gastric retention was evaluated using a dynamic human stomach-intestine (DHSI-IV) system, which realistically reproduces gastric peristalsis, pH variation, and emptying [26,27]. This work aims to clarify how polysaccharide reinforcement can stabilize swelling hydrogels and regulate transport, providing design principles for mechanically robust, food-grade gastric-retentive systems.

## 2. Results and Discussion

### 2.1. Effect of Curdlan Content on Swelling Stability in Simulated Gastric Conditions

**Figure 1:**
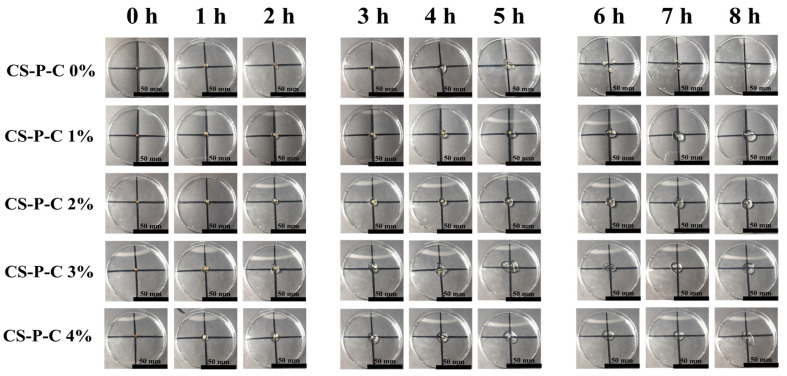
Macroscopic appearance of CS/CUR/PAAS hydrogels with different curdlan contents (0–4 wt.%) during 8 h immersion in simulated gastric fluid (SGF, pH 1.2).

Figure 1 presents the macroscopic morphological evolution of CS/CUR/PAAS hydrogels with varying curdlan contents (0–4 wt.%) during immersion in simulated gastric fluid (SGF, pH 1.2). All formulations initially exhibited a characteristic core–shell architecture, consisting of a dense chitosan-based core surrounded by a swollen, translucent outer layer formed upon acid-induced hydration. This swelling-induced core–shell morphology is consistent with previous reports on chitosan-based gastric-retentive hydrogels [16]. As digestion progressed, however, pronounced differences in swelling stability and structural integrity became evident, highlighting the critical role of CUR content in regulating swelling-driven gastric retention. Specifically, the CS-P-C 0% hydrogel exhibited the poorest structural stability. Although a relatively intact spherical morphology was maintained during the first 4 h of SGF exposure, progressive softening and fragmentation occurred thereafter, ultimately leading to partial disintegration (Figure 1). The hydrogels containing low (1%) or relatively high (3%) CUR contents were similarly unable to preserve well-defined shapes beyond 5 h, as evidenced by blurred boundaries and surface deformation. In contrast, excessive curdlan loading (CS-P-C 4%) resulted in pronounced spreading and loss of geometric integrity at later stages, suggesting that over-reinforcement disrupted the balance between swelling capacity and mechanical cohesion. Notably, the CS-P-C 2% hydrogel maintained a stable and intact morphology throughout the entire 8 h digestion period, indicating an optimal balance between swelling-induced expansion and mechanical resistance under acidic gastric conditions. This balance makes it an ideal carrier for achieving prolonged gastric retention and sustained drug release.

**Figure 2:**
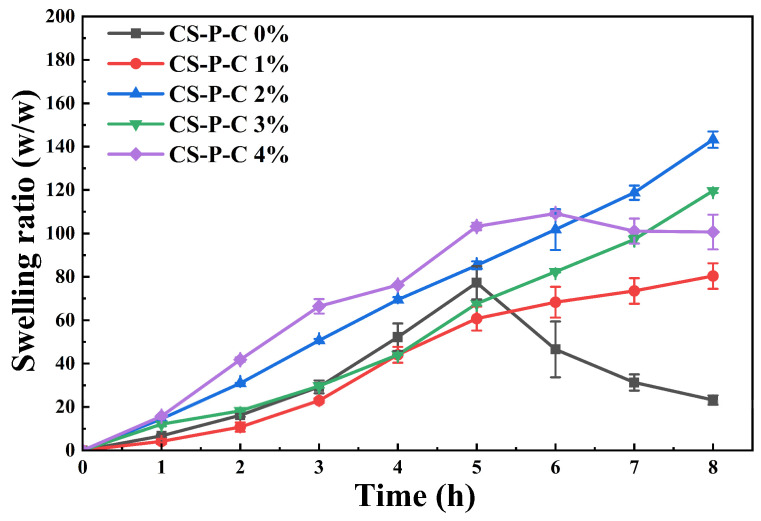
Swelling behavior of CS/CUR/PAAS hydrogels with different curdlan contents (0–4 wt.%) during incubation in simulated gastric fluid (SGF, pH 1.2), shown as the time-dependent evolution of the corresponding swelling ratios.

Figure 2 presents the swelling ratios of the hydrogels during incubation in SGF. All samples exhibited continuous water uptake during the first 5 h, reaching swelling ratios of at least 60-fold. At 8 h, swelling ratios exceeded 80-fold, with maximum values approaching 140-fold. The CS-P-C 0% hydrogel swelled rapidly to ~79-fold within 5 h but subsequently showed a sharp decline to ~20-fold between 5 and 8 h, consistent with the macroscopic disintegration observed in Figure 1. This behavior is attributed to the absence of curdlan reinforcement, resulting in a mechanically weak network that cannot withstand prolonged swelling-induced stress and gastric-like shear. Progressive network rupture and fragmentation under these conditions lead to loss of structural integrity, and the measured swelling ratio reflects only the remaining hydrogel mass rather than the original swollen structure. Incorporation of CUR effectively restrained excessive unfolding of surface polymer chains, thereby improving swelling stability. Although CS-P-C 4% achieved the fastest swelling rate (~110-fold within 5 h), partial degradation of the outer shell under shear resulted in a subsequent decrease, indicating insufficient mechanical robustness despite high swelling capacity. In contrast, CS-P-C 2% exhibited a uniquely balanced swelling profile, characterized by sustained, monotonic expansion and superior structural integrity. While its early-stage swelling ratio was slightly lower than that of CS-P-C 4%, it continued to increase steadily, reaching ~140-fold at 8 h without signs of mechanical collapse.

Collectively, these results demonstrate that an optimal CUR content enables partial decoupling of swelling capacity from mechanical failure, thereby ensuring stable size enlargement required for effective gastric retention [28]. Compared with previously reported gastric-retentive hydrogels, the CS-P-C system exhibits superior swelling stability under strongly acidic conditions. For example, previous studies have reported that a hydrogel based on sodium carboxymethyl cellulose (CMC), citric acid (CA) and hyaluronic acid (HA) (denoted as CMC-CA-HA) achieved a maximum swelling ratio of 6669 ± 1622% in diluted SGF (pH 2.1), while its swelling capacity decreased over time [29]. Similarly, a hydrogel with nearly 600-fold water absorption at pH 7.3, whereas swelling was limited to ~100-fold at pH 1.0 [2]. In contrast, the CS-P-C hydrogels developed here maintained continuous swelling and structural integrity in SGF (pH 1.2), highlighting their potential for biomedical and oral delivery applications requiring prolonged gastric residence.

### 2.2. Effect of Curdlan Content on Size Expansion in Simulated Gastric Conditions

**Figure 3:**
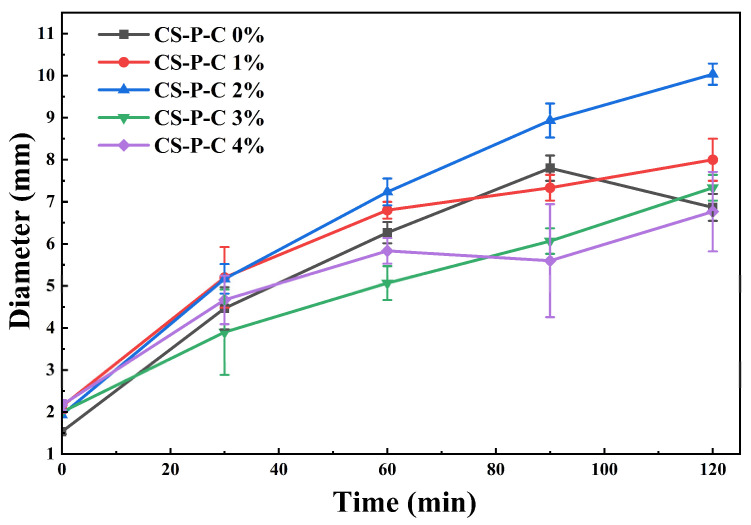
Swelling behavior of CS/CUR/PAAS hydrogels with different curdlan contents (0–4 wt.%) during incubation in simulated gastric fluid (SGF, pH 1.2), showing the time-dependent evolution of hydrogel bead diameter.

The swelling-induced size evolution of the hydrogel beads is shown in Figure 3. All formulations exhibited rapid diameter expansion during the initial swelling stage (within 2 h), reaching final diameters in the range of 6–10 mm. Notably, all hydrogels exceeded 3 mm in diameter within 30 min, surpassing the reported pyloric opening in humans (~2 mm) under fed conditions and thereby meeting the geometric requirement for gastric retention [30]. Despite this common early expansion, pronounced differences in the temporal evolution and stability of particle size were observed among formulations. For CS-P-C 0%, the diameter increased rapidly from approximately 1 mm to 7.5 mm within 90 min, followed by a reduction to ~6.5 mm at 120 min. This size decrease indicates mechanical weakening of the hydrogel matrix, likely caused by extensive protonation of chitosan chains and shear-induced surface erosion under acidic conditions, as previously reported for weakly crosslinked hydrogels [31]. In contrast, hydrogels containing CUR exhibited improved dimensional stability. The CS-P-C 1% formulation showed rapid early swelling (~225% increase within 60 min), followed by a slower expansion phase. Among all samples, CS-P-C 2% displayed the most favorable swelling behavior, expanding uniformly from an initial diameter of ~2 mm to ~10 mm within 120 min (approximately 400% increase). This final size exceeded that of the weakest-performing formulation (CS-P-C 4%) by ~3.5 mm, underscoring the effectiveness of moderate CUR incorporation in stabilizing swelling without inducing mechanical failure. Although CUR did not markedly increase intrinsic swelling capacity, its reinforcing role enhanced network integrity, enabling sustained water uptake and dimensional stability [32]. By comparison, CS-P-C 3% reached a lower final diameter (~8 mm) after rapid initial swelling, while CS-P-C 4% exhibited non-monotonic size evolution, characterized by a transient decrease at 90 min. This behavior is attributed to excessive outer-layer chain extension and shear-induced structural disruption, followed by renewed swelling driven by continued protonation of the internal network. Such competing effects ultimately promote progressive network disintegration, consistent with observations in other physically crosslinked hydrogel systems [33].

### 2.3. Mechanical Strength and Elastic Behavior of Curdlan-Reinforced Hydrogels

**Figure 4:**
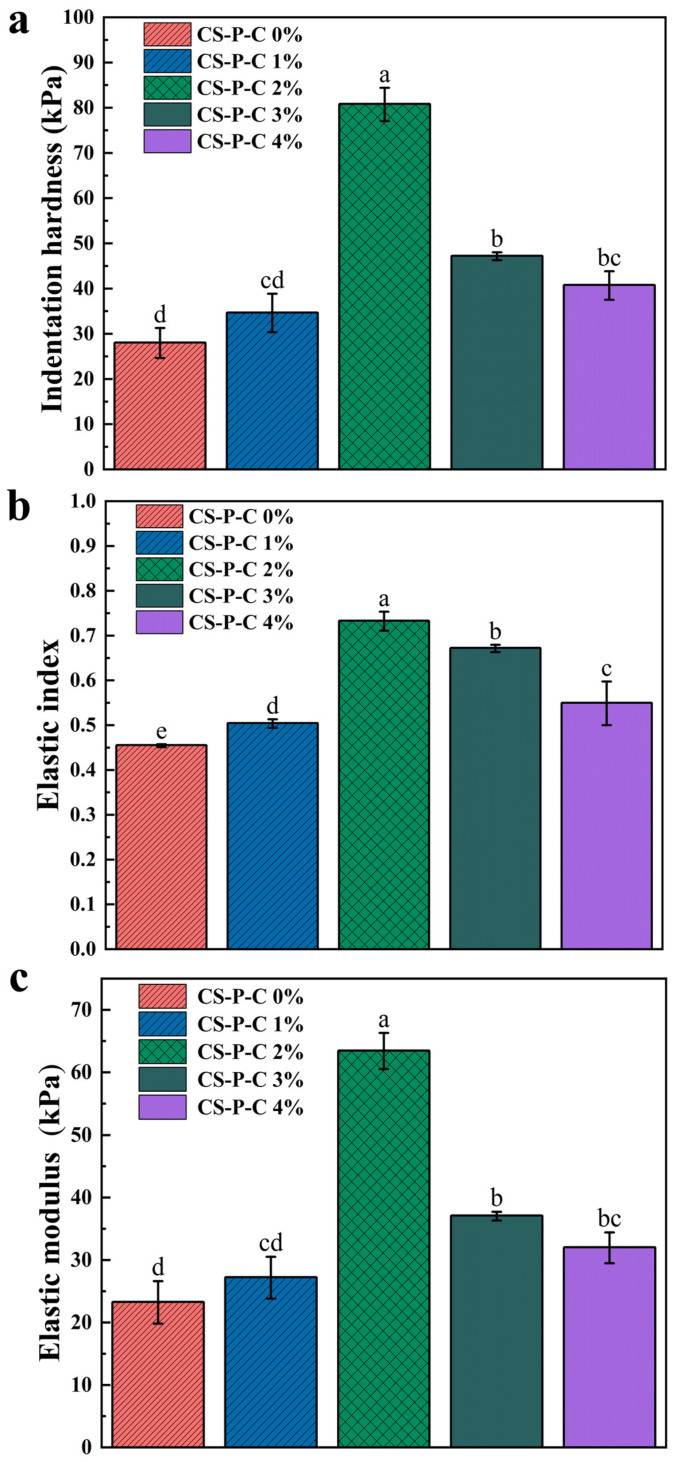
Mechanical properties of CS/CUR/PAAS hydrogels containing varying curdlan contents (0–4 wt.%) prior to drying, measured using a microstructure analyzer (MTA). (**a**) Indentation hardness; (**b**) Elasticity index; (**c**) Elastic modulus. Data are presented as the mean ± standard deviation (SD) (n = 3). Different letters in the panels indicate statistically significant differences (*p* < 0.05).

To ensure effective gastric retention, hydrogel systems must simultaneously resist compressive deformation and withstand repetitive mechanical stresses arising from gastric motility. Accordingly, the mechanical properties of CS/CUR/PAAS hydrogels were evaluated in their hydrated state prior to drying, corresponding to the conditions experienced during gastric residence. Figure 4a presents the indentation hardness (*H*), which reflects resistance to surface deformation and penetration. The hardness values followed the order: CS-P-C 2% (80 kPa) > CS-P-C 3% (47 kPa) > CS-P-C 4% (40 kPa) > CS-P-C 1% (34 kPa) > CS-P-C 0% (27 kPa), with significant differences (*p* < 0.05). Incorporation of CUR markedly enhanced hydrogel hardness, with CS-P-C 2% exhibiting an approximately 196% increase relative to the CUR-free control. This improvement indicates that CUR contributes to the formation of a denser and more mechanically robust network, thereby enhancing resistance to indentation and surface damage [16].

In addition to strength, gastric-retentive hydrogels must exhibit sufficient elasticity to undergo reversible deformation and recover their original shape under cyclic gastric stresses. Figure 4b shows the elastic index (*I_E_*), which characterizes elastic recovery and network stability. CS-P-C 0% exhibited the lowest elasticity (*I_E_* = 0.45), while a modest increase was observed for CS-P-C 1% (*I_E_* = 0.50). The highest *I_E_* was achieved at 2% CUR (*I_E_* = 0.73), indicating optimal elastic recovery at this concentration. Meanwhile, the elastic modulus of this formulation was higher than that reported for a chitosan-based hydrogel by Wang et al. [16] (0.67 kPa), suggesting that curdlan incorporation contributes to improved mechanical performance. Further increases in CUR content resulted in a decline in elasticity, with *I_E_* values decreasing to 0.67 and 0.54 for CS-P-C 3% and CS-P-C 4%, respectively. This observation is consistent with previous reports [18] that moderate CUR incorporation enhances elastic resilience, whereas excessive CUR restricts network flexibility, likely due to increased network rigidity and reduced polymer chain mobility.

The elastic modulus (E*) shown in Figure 4c further reflects resistance to elastic deformation [34]. The elastic moduli followed the order: CS-P-C 2% (63 kPa) > CS-P-C 3% (37 kPa) > CS-P-C 4% (31 kPa) > CS-P-C 1% (27 kPa) > CS-P-C 0% (23 kPa), with significant differences (*p* < 0.05) observed among CS-P-C 0%, CS-P-C 2%, and CS-P-C 3%. Notably, the elastic modulus of CS-P-C 2% exceeded the reported maximum human gastric pressure (10–13 kPa) by a wide margin [35], indicating that CUR incorporation effectively endows the hydrogel with sufficient mechanical robustness to withstand physiological gastric stresses. However, further increases in CUR content did not yield additional mechanical benefits. Instead, a decline in modulus was observed beyond 2% CUR, likely due to curdlan aggregation and reduced solubility at higher concentrations, which compromises effective network formation [36].

### 2.4. Gastric Retention Characteristics in the DHSI-IV System

**Figure 5:**
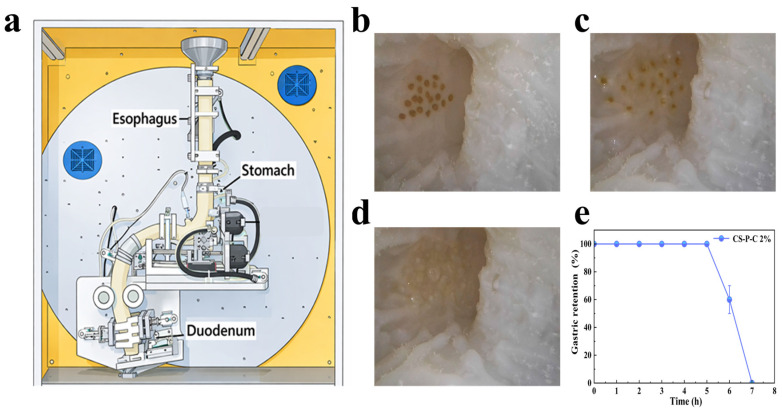
Dynamic gastric retention behavior of the CS-P-C 2% hydrogels evaluated using the DHSI-IV (NERDT) system. (**a**) Schematic illustration of the DHSI-IV system. (**b**–**d**) Representative images showing the position and morphology of the hydrogels within the gastric chamber at 1, 4, and 7 h, respectively. (**e**) Quantitative gastric retention profile of the hydrogels under simulated fasting conditions over 7 h (mean ± SD, n = 3).

As shown in Figure 5, the CS-P-C 2% hydrogel exhibited pronounced gastric retention behavior in the DHSI-IV system. Upon introduction into the stomach model, the hydrogel rapidly swelled while maintaining an intact spherical morphology during the initial digestion phase. Throughout the first 5 h, the hydrogel remained structurally stable within the gastric chamber (Figure 5b,c), with approximately 60% of the initial mass retained at 6 h (Figure 5e). This behavior can be attributed to the combination of swelling-induced size enlargement and sufficient mechanical strength, which together enabled the hydrogel to resist fluid shear forces and compressive stresses generated by simulated gastric peristalsis. After approximately 5 h, gradual emptying of the hydrogel was observed (Figure 5d), indicating a time-dependent loss of retention capability. This transition is consistent with the rheological results as presented in Section 2.6, which showed a progressive decrease in the storage modulus (G′) of the hydrogel under acidic conditions. The continuous softening of the network reduced its resistance to deformation, eventually allowing passage through the pyloric opening under dynamic conditions [26].

Importantly, the gastric retention performance of the present system compares favorably with previously reported metformin delivery platforms. Floating systems, such as microballoon-based formulations, can prolong gastric residence time; however, their retention strongly depends on gastric fluid dynamics and is prone to failure under variable motility conditions due to density-dependent buoyancy loss [37]. Mucoadhesive systems improve initial gastric residence by adhesion to the mucosal layer, but their performance is limited by mucus turnover and shear-induced detachment in dynamic gastric environments, typically restricting effective retention to a few hours [38]. In contrast, swelling-controlled hydrogel systems provide geometry-driven retention but often suffer from mechanical weakening or structural collapse under prolonged acidic exposure, particularly for highly water-soluble drugs such as metformin hydrochloride [35].

In comparison, the CS-P-C hydrogel developed in this study integrates swelling-induced expansion, curdlan-reinforced interpenetrating network stabilization, and shear-resistant structural integrity, enabling a more robust retention mechanism under physiologically relevant dynamic gastric conditions. The effective retention window of approximately 5 h closely matches the primary release phase of metformin hydrochloride, ensuring sufficient residence time for sustained drug release while avoiding excessive gastric accumulation [23,26]. Notably, no pyloric obstruction, sudden mass discharge, or intermittent blockage was observed throughout the experiment, indicating a stable and reproducible gastric emptying behavior.

### 2.5. Microstructure of the Hydrogels During Gastric Digestion

**Figure 6:**
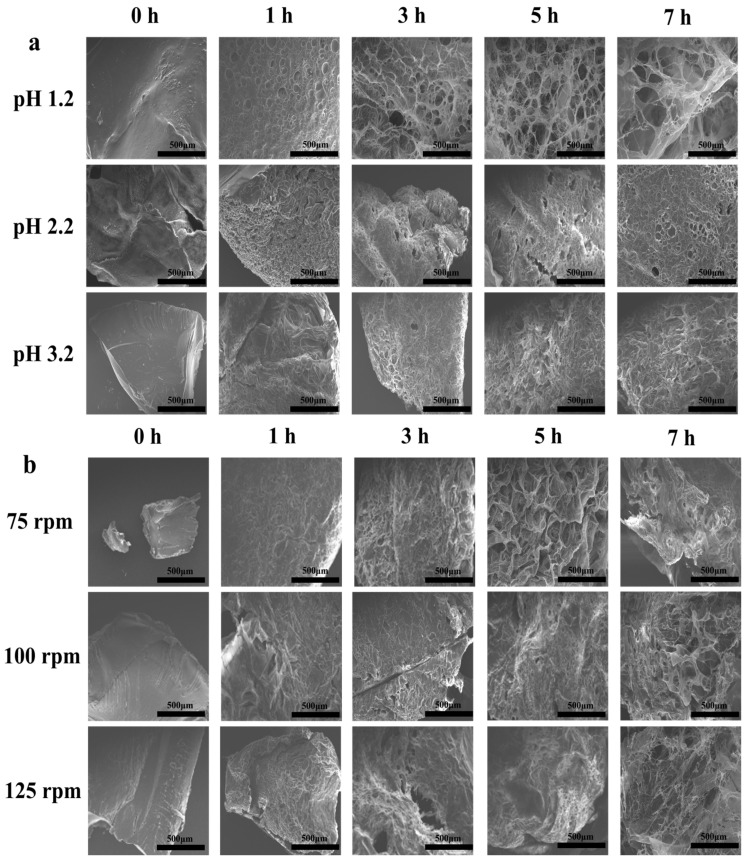
SEM images of chitosan-based CS-P-C 2% hydrogels after gastric digestion for 0, 1, 3, 5, and 7 h. (**a**) Microstructural evolution under different gastric acidity conditions (pH 1.2, 2.2, and 3.2), with agitation speed fixed at 100 rpm. (**b**) Microstructural evolution under different gastric motility intensities (75, 100, and 125 rpm), with pH fixed at 2.2.

Figure 6 presents scanning electron microscopy (SEM) images of the CS-P-C 2% hydrogel after incubation in SGF for 0, 1, 3, 5, and 7 h under varying gastric pH values (1.2, 2.2, and 3.2) and motility intensities (75, 100, and 125 rpm). These images reveal how chemical (pH) and mechanical (agitation) stimuli jointly regulate the microstructural evolution of the hydrogel during gastric digestion. As shown in Figure 6a, the hydrogel initially exhibited a dense and compact morphology under all pH conditions. With increasing digestion time, progressive pore formation and network expansion were observed, with the extent of structural opening strongly dependent on gastric acidity. At pH 1.2, rapid protonation of chitosan chains led to pronounced swelling and the formation of large, interconnected pores as early as 1–3 h, which further expanded between 3 and 7 h. In contrast, at pH 2.2 and 3.2, pore development occurred more gradually, and the hydrogel retained a comparatively denser network throughout digestion. These observations indicate that lower pH accelerates polymer chain relaxation and water uptake, thereby enlarging diffusion pathways but potentially weakening structural integrity. An intermediate acidic environment therefore provides a more balanced microstructure, offering sufficient porosity for drug diffusion while maintaining network cohesion [39,40].

The influence of mechanical agitation on hydrogel microstructure is illustrated in Figure 6b. Increasing agitation speed from 75 to 125 rpm progressively enhanced surface erosion and pore formation at identical digestion times. At 3 h, distinct diffusion channels were evident at 125 rpm, whereas samples exposed to lower agitation remained relatively compact. After 7 h, high motility conditions resulted in a fully developed three-dimensional porous network, while hydrogels subjected to mild agitation preserved a more robust and less open structure. Compared with pH effects, gastric motility exerted a secondary but non-negligible role, primarily accelerating pore formation rather than fundamentally altering the network architecture.

To quantitatively support these observations, we performed image analysis of the SEM images to determine the mean pore radius under the different experimental conditions. As shown in Table S1 of Supporting Material, the mean pore radius increased over time under all conditions, with the most significant pore expansion observed at pH 1.2. For example, at pH 1.2—100 rpm, the mean pore radius increased from 14.94 ± 6.82 μm at 1 h to 64.91 ± 38.55 μm at 7 h. At pH 2.2—100 rpm, the increase was more moderate, with a mean pore radius of 17.49 ± 5.30 μm at 1 h and 34.28 ± 5.89 μm at 7 h. These results quantitatively confirm that the lower pH conditions led to a more rapid and pronounced expansion of pore size, consistent with the SEM images. Overall, SEM analysis demonstrates that gastric pH is the dominant factor governing microstructural evolution of the CS-P-C 2% hydrogel, while gastric motility intensity modulates the rate of structural opening. The combined chemical-mechanical regulation of porosity is consistent with the observed gastric retention behavior (Figure 5), confirming the relevance of physiologically realistic pH and motility conditions in evaluating hydrogel performance.

### 2.6. Storage and Loss Moduli of the Hydrogels During Gastric Digestion

**Figure 7:**
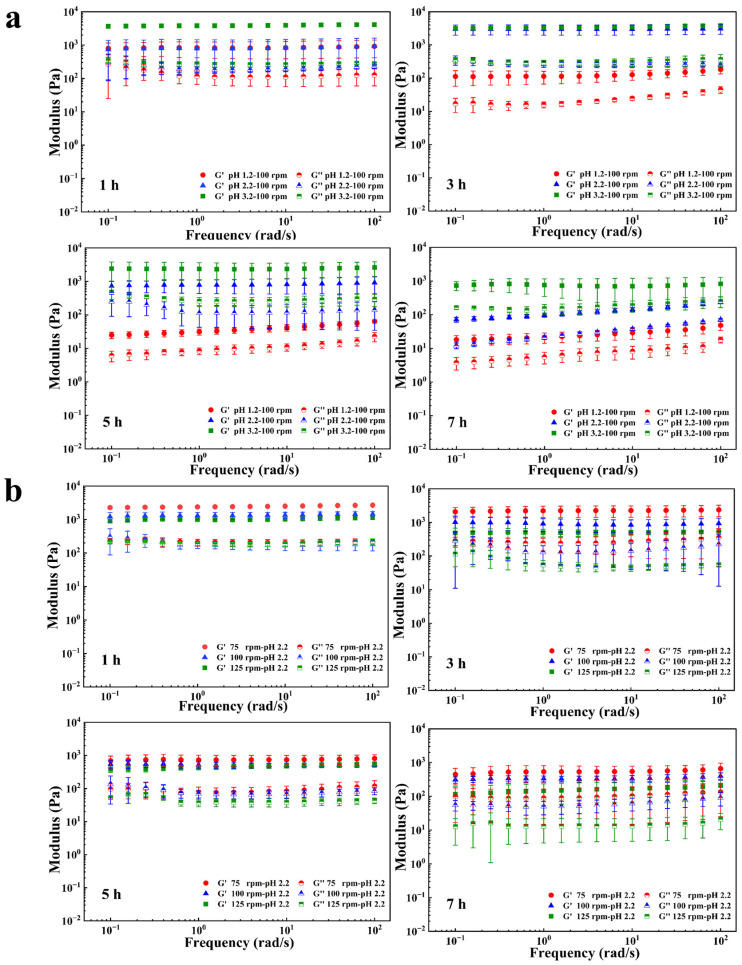
Evolution of the storage modulus (G′) and loss modulus (G″) of the chitosan-based CS-P-C 2% hydrogels during simulated gastric digestion at different time points (1, 3, 5, and 7 h). (**a**) Viscoelastic response of the hydrogels under different gastric acidity conditions (pH 1.2, 2.2, and 3.2). (**b**) Viscoelastic response of the hydrogel under different simulated gastric motility intensities, represented by agitation speeds of 75, 100, and 125 rpm. Data are expressed as mean ± standard deviation (SD) (n = 3).

As shown in Figure 7, the storage modulus (G′) remained consistently higher than the loss modulus (G″) throughout the digestion period, indicating that the hydrogel exhibited predominantly elastic behavior within the tested deformation range. This dominance of G′ over G″ confirms the presence of a well-developed gel network capable of sustaining elastic deformation while limiting viscous energy dissipation under oscillatory stress [41]. The acidic environment exerted a pronounced influence on the viscoelastic properties of the hydrogel (Figure 7a). Under strongly acidic conditions (pH 1.2), G′ decreased sharply from approximately 1000 Pa at 1 h to about 100 Pa at 3 h, followed by stabilization near 80 Pa after 7 h. A similar trend was observed for G″, which declined rapidly from ~130 Pa to ~20 Pa over the same period. In contrast, hydrogels exposed to moderately acidic environments (pH 2.2) maintained substantially higher moduli during the early digestion stage, with G′ remaining above 1000 Pa at 1 h and decreasing to approximately 231 Pa only after 5–7 h. Correspondingly, G″ decreased from 269 Pa at 1 h to around 10 Pa after 5 h. Under weakly acidic conditions (pH 3.2), the hydrogel showed the highest resistance to mechanical softening, with G′ remaining above 1000 Pa during the first 3 h and still exceeding 750 Pa at 7 h, while G″ remained above 150 Pa throughout digestion. These results demonstrate that increasing acidity accelerates network relaxation and mechanical degradation, in agreement with the observed microstructural evolution and drug release behavior, highlighting pH as a dominant regulator of hydrogel stability [42].

To further quantify the viscoelastic balance, the loss factor (tan δ = G″/G′) was calculated at a fixed frequency of 1 Hz. Although human gastric peristalsis typically occurs at a lower frequency (~0.05 Hz) [43,44], 1 Hz is commonly adopted in hydrogel rheology to ensure measurements within the linear viscoelastic region and to enable comparison with literature data. Under all digestion conditions, tan δ remained below 0.3, confirming that elastic behavior predominated (tan δ < 1). Under strongly acidic conditions (pH 1.2), tan δ increased from approximately 0.15 at 1 h to approximately 0.20 at 7 h, indicating progressive network relaxation and an increasing viscous contribution. Under weakly acidic conditions (pH 3.2), tan δ increased from approximately 0.07 at 1 h to approximately 0.20 at 7 h, reflecting a slower structural evolution. Detailed values are provided in Table S2 of the Supplementary Materials.

In comparison, gastric motility intensity exerted a secondary but discernible effect on the viscoelastic behavior of the hydrogel (Figure 7b), which is consistent with the SEM observations (Figure 6). Within the first 5 h of digestion, variations in agitation speed (75–125 rpm) produced no substantial differences in G′, which remained above 500 Pa in all cases. However, higher shear intensities accelerated the decline of both G′ and G″ at later stages. At 125 rpm, G″ decreased from approximately 170 Pa at 1 h to 40 Pa by 5 h, accompanied by a more pronounced reduction in G′. After 7 h, the hydrogel subjected to the highest shear exhibited significantly lower G′ (95 Pa) and G″ (20 Pa) than those exposed to lower agitation speeds, although these values remained higher than those measured under strongly acidic conditions.

To further the shear effect on viscoelastic balance, tan δ was further analyzed. Under all agitation conditions, tan δ remained below 0.25 (Table S2), confirming the persistence of elastic-dominant behavior. At 125 rpm, tan δ decreased from approximately 0.19 at 1 h to 0.09 at 7 h, suggesting that although shear accelerates structural erosion, the remaining network becomes relatively more elastic, likely due to preferential loss of loosely associated polymer regions. In contrast, at 75 rpm, tan δ increased from approximately 0.09 to 0.17 over the same period, indicating gradual accumulation of viscous contributions under milder mechanical perturbation. These results suggest that shear modulates the pathway of structural evolution rather than inducing a true elastic-to-viscous transition. No crossover frequency was observed under any shear condition, further confirming the absence of a gel-to-sol transition. This shear-induced softening is attributed to enhanced water uptake and mechanical perturbation, which promote polymer chain relaxation, network expansion, and partial dissociation of the gel structure [45].

### 2.7. pH- and Shear-Regulated Metformin Release Kinetics

**Figure 8:**
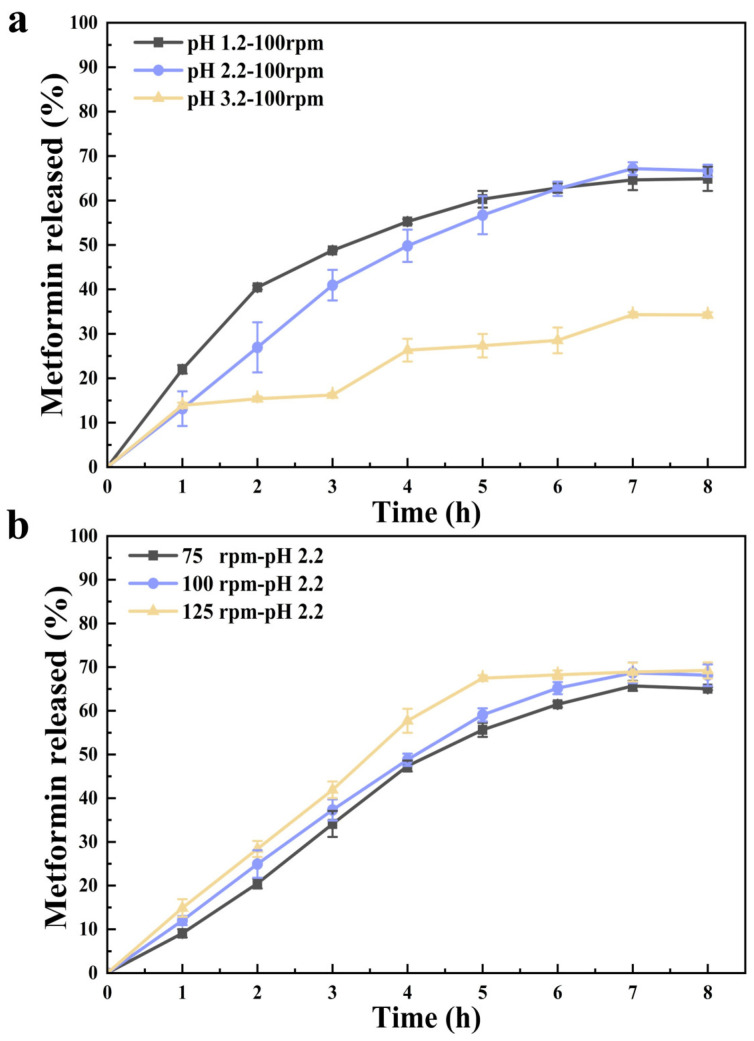
In vitro release profiles of metformin hydrochloride from CS-P-C 2% hydrogels during simulated gastric digestion. (**a**) Effect of gastric acidity on drug release at pH 1.2, 2.2, and 3.2 with agitation speed fixed at 100 rpm. (**b**) Effect of simulated gastric motility intensity on drug release at agitation speeds of 75, 100, and 125 rpm with pH maintained at 2.2. Data are expressed as mean ± standard deviation (SD) (n = 3).

The influence of gastric acidity on metformin hydrochloride release is shown in Figure 8a. A clear pH-dependent release behavior was observed. Under strongly acidic conditions (pH 1.2), a pronounced burst release occurred within the first hour, reaching a cumulative release of approximately 22%, significantly higher than that observed at pH 2.2 and 3.2 (≈13%, *p* < 0.05). This behavior is attributed to extensive protonation of chitosan chains at low pH, which induces rapid chain extension and weakens the constraining effect of the physically cross-linked CUR network, thereby accelerating drug diffusion [3,46]. Between 1 and 4 h, the release rate under pH 1.2 increased sharply from 22% to 55%, followed by a slower release phase, reaching a plateau of ~66% at 7 h. This transition reflects a shift from initial swelling-driven diffusion to a more diffusion-limited regime as the network structure stabilizes despite ongoing relaxation. At pH 2.2, drug release proceeded in a more controlled manner. The cumulative release increased gradually from 13% at 1 h to approximately 40% at 3 h, followed by sustained release up to ~67% at 7 h. This behavior reflects a balance between sufficient protonation-induced swelling and the mechanical reinforcement provided by CUR and sodium tripolyphosphate (STPP) crosslinking, which together promote prolonged release [47]. In contrast, under weakly acidic conditions (pH 3.2), insufficient protonation limited polymer chain extension, resulting in restricted swelling and a stepwise release profile. After an initial release of ~13% at 1 h, minimal additional release occurred until 3 h, and the final cumulative release reached only ~34% at 7 h. Although this condition exhibited the slowest release rate, the limited total release indicates inadequate activation of the hydrogel network for effective drug delivery.

To quantitatively describe the release mechanism, the release profiles were fitted using the Higuchi model [48]. Across all pH and shear conditions except pH 3.2, the model provided an excellent fit with high correlation coefficients (R^2^ = 0.987), indicating that metformin release is predominantly governed by Fickian diffusion from a swelling-controlled matrix. The deviation observed at pH 3.2 further supports that insufficient swelling disrupts the assumptions of the Higuchi model, leading to non-ideal diffusion behavior.

The effect of gastric motility intensity on metformin release is presented in Figure 8b. Increasing agitation speed enhanced the drug release rate, consistent with intensified disruption of the diffusion boundary layer and shear-induced micro-defects within the hydrogel network [49]. Under high shear conditions (125 rpm), approximately 30% of metformin was released within the first 2 h, and near-equilibrium release was achieved by 5 h. At lower agitation speeds (75 and 100 rpm), the release profiles were similar, with ~48% released within 4 h followed by a slower release phase, reaching ~68% at 7 h. Notably, despite the enhanced release under high shear, the overall modulation effect remained weaker than that induced by strong acidity, further confirming that hydrogel swelling and network relaxation are primarily pH-governed processes. Overall, these results demonstrate that metformin release from the CS-P-C 2% hydrogel is jointly regulated by gastric pH and motility, with acidity serving as the dominant trigger and shear acting as an auxiliary accelerator. This dual-responsive behavior aligns well with physiological gastric conditions and underpins the rational design of mechanically robust, pH-adaptive gastric-retentive delivery systems.

## 3. Conclusions

In this study, a curdlan-reinforced chitosan-based composite hydrogel was developed and systematically evaluated as a gastric-retentive carrier for metformin hydrochloride under dynamic gastric conditions. Among the investigated formulations with varying curdlan contents (0–4 wt.%), CS-P-C 2% (containing 2 wt.% curdlan) exhibited the most favorable balance between swelling capacity and mechanical stability. This formulation showed the highest indentation hardness (≈80 kPa), elastic index (0.73), and elastic modulus (≈63 kPa), values well above reported physiological gastric pressures (10–13 kPa), enabling the hydrogel to withstand gastric compression and shear without premature failure. As a result, the CS-P-C 2% hydrogel maintained effective gastric retention for approximately 5 h under fasting conditions in the DHSI-IV system, with about 60% of the hydrogel retained at 6 h, followed by gradual and unobstructed emptying. Microstructural and rheological analyses revealed that gastric pH plays a dominant role in regulating network expansion, pore formation, and viscoelastic relaxation, whereas gastric motility intensity exerts a secondary but measurable influence. These structure-property changes directly governed drug release behavior. Under strongly acidic conditions (pH 1.2), a burst release of ~22% occurred within 1 h, followed by accelerated release to ~66% at 7 h, whereas under weakly acidic conditions (pH 3.2), cumulative release was limited to ~34%. Increased shear further accelerated release, particularly at higher agitation rates. Overall, moderate curdlan incorporation enables partial decoupling of swelling from mechanical failure in chitosan-based hydrogels, supporting stable gastric retention and predictable release behavior under physiologically relevant conditions. This work provides a rational framework for designing mechanically robust, pH-responsive gastric-retentive hydrogel systems for oral delivery of highly water-soluble drugs. Future work will focus on validating in vitro–in vivo correlations, refining hydrogel composition to enable programmable residence time and release kinetics, and further evaluating biological safety through in vitro cytotoxicity assays and in vivo gastric mucosal irritation studies. Consideration of processing consistency and material quality will also be important for potential scale-up and pharmaceutical application.

## 4. Materials and Methods

### 4.1. Materials

Chitosan (CS; viscosity ≥ 400 mPa·s, measured in 0.1% (*v*/*v*) acetic acid at 20 °C; degree of deacetylation ≥ 95%, as specified by the supplier) was obtained from Shanghai Macklin Biochemical Technology Co., Ltd. (Shanghai, China). According to the supplier’s specification, the weight-average molecular weight of chitosan is approximately 3.5 × 10^5^ g/mol, corresponding to a high-molecular-weight grade. Curdlan (CUR; biochemical reagent grade; Product No. R005334) was purchased from Shanghai Yien Chemical Technology Co., Ltd. (Shanghai, China). According to the supplier, the weight-average molecular weight of curdlan is approximately 5.0 × 10^5^ g/mol in aqueous NaOH (0.5 mol/L), as determined by size exclusion chromatography coupled with multi-angle laser light scattering (SEC-MALLS). Curdlan is a linear, non-branching polysaccharide composed of glucose residues linked by β-(1 → 3) glycosidic bonds and is known to form thermally induced gels through the aggregation of triple-helical structures [17]. Sodium polyacrylate (PAAS; weight-average molecular weight ≈ 3.0 × 10^6^ g/mol, as determined by viscometry; Product No. 01284004) and sodium tripolyphosphate (STPP; Product No. 238503) were supplied by Shanghai Titan Scientific Co., Ltd. (Shanghai, China). Metformin hydrochloride (MET; Product No. MJ10199; purity ≥ 98%, as determined by HPLC) was purchased from Zancheng (Tianjin) Technology Co., Ltd. (Tianjin, China). All other chemicals and reagents were of analytical grade and used as received without further purification. Ultrapure water (Milli-Q; resistivity ≥ 18.2 MΩ·cm) produced using a Heal Force water purification system was used throughout all experiments.

### 4.2. Preparation of Core–Shell CS/CUR/PAAS Hydrogels

**Figure 9:**
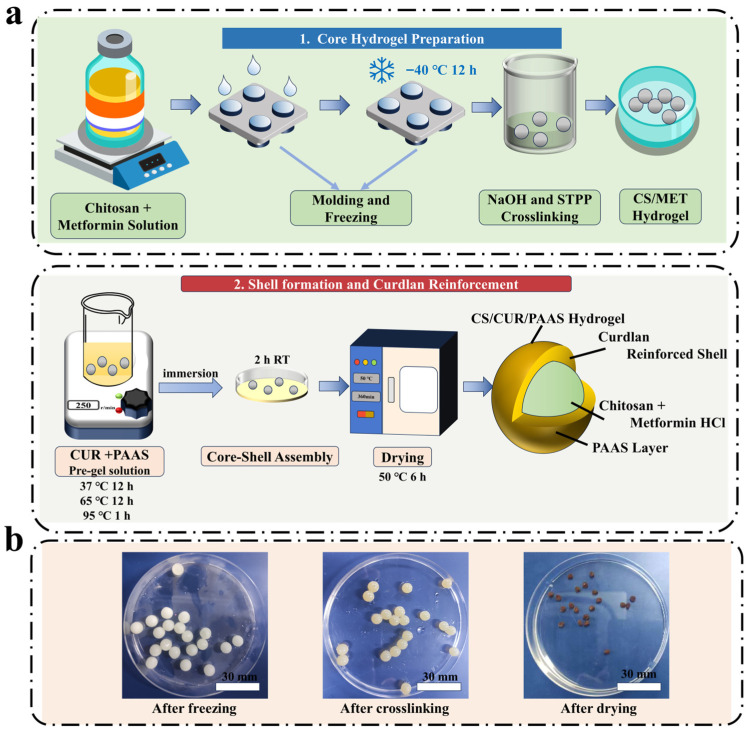
Schematic illustration of the fabrication of core–shell CS/CUR/PAAS hydrogels. (**a**) Stepwise preparation of the chitosan-based hydrogel core, including drug loading, freezing-induced gelation, alkali neutralization, and ionic crosslinking. (**b**) Representative photographs of the hydrogel at key fabrication stages, from initial core formation to the final curdlan-reinforced core–shell structure.

Core–shell chitosan/curdlan/sodium polyacrylate (CS/CUR/PAAS) hydrogels were prepared via a sequential gelation and reinforcement strategy, adapted from following the method of Wang et al. [16]. The fabrication process comprised two main steps: (i) formation of spherical, drug-loaded chitosan hydrogel cores through freezing-induced gelation combined with alkali neutralization and ionic crosslinking, and (ii) construction of a mechanically reinforced outer shell via curdlan-assisted composite gelation. A schematic overview is shown in Figure 9.

#### 4.2.1. Preparation of Chitosan-Based Hydrogel Cores

Chitosan solution (5 wt.%) was prepared by dissolving 5 g of chitosan in 94 g of 0.2 mol/L acetic acid under magnetic stirring (250 rpm) at 25 ± 1 °C for 48 h, followed by standing for 6 h to remove air bubbles. Metformin hydrochloride (1 g) was added and stirred for 30 min to obtain a homogeneous drug-loaded solution. Aliquots (≈0.2 mL) of this solution were dispensed into hemispherical cavities (7.5 mm diameter). To minimize pore heterogeneity, all samples were placed on a pre-cooled metal plate and frozen at −40 °C for 12 h in a static environment without forced convection, enabling directional bottom-to-top heat transfer and controlled ice crystal growth. The frozen beads were transferred into 0.1 mol/L NaOH solution and gently stirred for 5 min to neutralize residual acetic acid and stabilize the network [50]. After rinsing with deionized water until neutral pH, the beads were immersed in 1 wt.% STPP solution for 30 s to introduce ionic crosslinking, then extensively washed with deionized water. Washing steps were performed after each crosslinking stage to remove residual reagents and surface-associated free drug, reducing initial burst release. The resulting CS/MET hydrogel cores were used immediately for shell formation.

#### 4.2.2. Shell Formation and Curdlan Reinforcement

CUR and PAAS were dispersed in 0.01 mol/L NaOH solution at the desired concentrations (Table 1) and stirred at 600 rpm for 12 h at 25 °C to ensure complete hydration. The dispersion was heated to 65 °C with stirring at 300 rpm for 12 h, then heated to 95 °C for 1 h to induce irreversible thermal gelation of curdlan [17]. All heating steps were performed using a constant-temperature magnetic stirrer (±1 °C accuracy) to ensure consistent thermal conditions. After cooling to room temperature, the semi-fluid CUR/PAAS matrix was used for shell formation.

**Figure 10:**
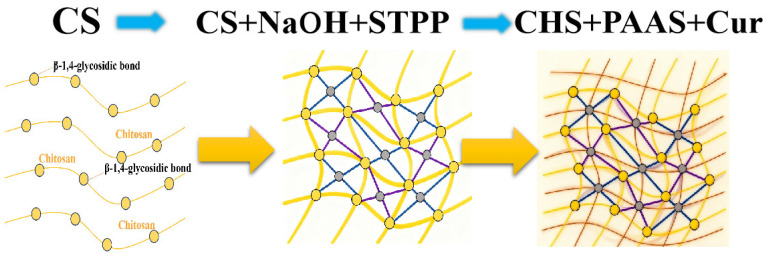
Schematic illustration of the sequential crosslinking and interpenetrating network formation in CS/CUR/PAAS hydrogels. Chitosan forms the primary network via NaOH neutralization and STPP ionic crosslinking, followed by curdlan reinforcement (β-(1 → 3) triple-helix network) and PAAS incorporation, yielding a mechanically stable and pH-responsive structure.

The preformed CS/MET hydrogel cores were immersed in the CUR/PAAS solution and allowed to undergo interfacial crosslinking and shell formation for 2 h at room temperature (25 ± 1 °C), resulting in the formation of a core–shell structure. During this process, curdlan underwent thermal gelation to form a rigid β-(1 → 3)-glucan network, which interpenetrated the outer chitosan layer and provided mechanical reinforcement. Meanwhile, PAAS functioned as a highly swellable polyelectrolyte, contributing to osmotic swelling and pH-responsive behavior of the composite hydrogel. The sequential crosslinking mechanisms, including primary network formation via NaOH neutralization and STPP ionic crosslinking, followed by secondary reinforcement through CUR/PAAS incorporation, are schematically illustrated in Figure 10.

After shell formation, the composite core–shell hydrogels were removed and dried at 50 °C for 6 h. Hydrogels with different curdlan concentrations (0–4 wt.%) were prepared by adjusting the CUR concentration in the shell formulation while keeping all other parameters constant. The resulting samples were denoted as CS-P-C 0%, CS-P-C 1%, CS-P-C 2%, CS-P-C 3%, and CS-P-C 4%. All hydrogels prepared in this study were subjected to at least three independent replicates (n = 3); the detailed formulations are summarized in Table 1.

### 4.3. Evaluation of Swelling Behavior and Size Evolution in Simulated Gastric Fluids

The swelling behavior and size evolution of CS/CUR/PAAS core–shell hydrogels (CS-P-C 0%, 1%, 2%, 3%, and 4%) were evaluated in simulated gastric fluid (SGF) to assess their expansion characteristics and gastric retention potential under acidic conditions. SGF (pH 1.2) was prepared according to Brodkorb et al. [51] with slight modifications using hydrochloric acid solution adjusted to pH 1.2. All experiments were conducted at 37 ± 0.5 °C to simulate physiological gastric temperature.

Dried hydrogel samples were accurately weighed to obtain the initial dry weight ($W_{0}$) and then immersed in 60 mL of SGF under static conditions. Swelling experiments were carried out for 8 h. At predetermined time intervals (every 1 h), the hydrogels were removed from the medium, gently blotted with filter paper to remove excess surface liquid, and immediately weighed to determine the swollen weight ($W_{t}$). Digital photographs were taken at each time point to record morphological changes and size evolution. All measurements were performed in triplicate.

The swelling ratio was calculated as follows [52]:

(1) $$\mathrm{Swelling}\;\mathrm{ratio}=\frac{W_{t}-W_{0}}{W_{0}}$$

where $W_{0}$ is the initial weight of the dried hydrogels (mg) and $W_{t}$ is the weight of the swollen hydrogels (mg) at time *t*.

In parallel, hydrogel diameter was measured using a digital caliper at 30 min intervals to quantitatively assess size expansion during swelling. The swollen diameters were compared with the reported pyloric opening size (approximately 2 mm under fed conditions) to evaluate the ability of the hydrogels to resist gastric emptying via size enlargement [53]. Throughout the swelling process, changes in shape integrity, surface morphology, and structural stability were recorded. Any deformation, cracking, or surface erosion was noted to elucidate the role of curdlan content in regulating swelling-induced dimensional stability under acidic conditions.

### 4.4. Micro-Indentation Mechanical Analysis

**Figure 11:**
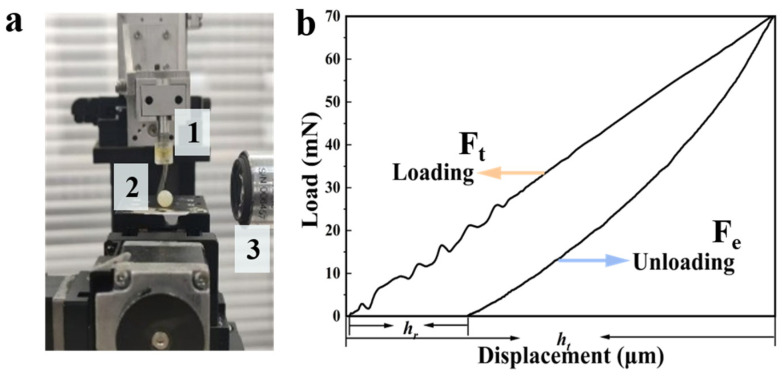
Schematic illustration of the microstructure analyzer (MTA). (**a**) Diagram illustrating the components and working principle of the MTA. (1) Probe, (2) hydrogel sample, and (3) camera. (**b**) Representative indentation loading-unloading curve, from which the maximum penetration depth (*h_t_*) and the residual plastic depth (*h_r_*) were obtained.

The mechanical properties of the CS/CUR/PAAS hydrogels were evaluated using a Microstructure Analyzer (MTA) with a displacement-controlled indentation system, as described by Lv et al. [54]. Indentation measurements were performed using a cylindrical probe operating at a frequency of 100 Hz, an indentation speed of 100 μm/s, and a maximum indentation depth of 1 mm. During testing, the instantaneous load applied to the hydrogel was continuously recorded by a high-resolution force sensor, enabling real-time acquisition of force-displacement data throughout the indentation process (Figure 11a). Cyclic indentation tests were conducted, in which the probe was driven into the hydrogel at a constant speed and then returned to its initial position after each loading cycle. The resulting loading-unloading force-displacement curves were used to quantify key micromechanical parameters, including indentation hardness, elastic modulus, and elasticity index. A representative indentation compliance curve is shown in Figure 11b.

Indentation hardness (*H*, kPa), describing the resistance of the hydrogel to localized deformation, was calculated from the maximum applied force as [54]:

(2) $$H=\frac{F}{A}=\frac{F}{\pi a^{2}}$$

where *F* is the applied load (mN), *A* is the contact area (mm^2^), and *a* is the radius of the cylindrical indenter (mm).

The elastic response of the hydrogel during indentation was analyzed using Hertzian contact theory. The load–displacement relationship is described by Lv et al. [54]:

(3) $$F\;=\;\frac{2E}{1-\nu^{2}}\;\times \;\mathrm{ah}\Pi$$

(4) $$E^{*}=\frac{E}{1\;-\;\nu^{2}}$$

where *E* and *v* represent Young’s modulus (kPa) and Poisson’s ratio, respectively; E* is the composite elastic modulus (kPa) reflecting the combined mechanical response of the hydrogel’s network under deformation, and *Π* denotes a dimensionless function that depends only on *a*/*t*_0_, where *a* is the contact radius and *t*_0_ is the specimen height.

The elasticity index (*I_E_*) was calculated to evaluate the relative contributions of elastic and plastic deformation of the hydrogels during indentation. It is defined as the ratio of elastic work to total work [54]:

(5) $$I_{E}=\frac{W_{e}}{W_{t}}$$

where *W_e_* represents the elastic work of indentation (the area under the unloading curve), and *W_t_* is the total work (the area under the loading curve). The total work and elastic indentation work were obtained by integrating the force-displacement curves during the loading and unloading phases [54]:

(6) $$W_{e}=\int_{0}^{h_{t}}F_{e}\mathrm{dh}$$

(7) $$W_{t}=\int_{0}^{h_{t}}F_{e}\mathrm{dh}$$

where *F_e_* is the elastic force, and *F_t_* is loading force. *h_t_* is the maximum penetration depth (mm).

### 4.5. Gastric Retention Evaluation Using the DHSI-IV System

Based on its favorable mechanical performance and swelling behavior, the CS-P-C 2% hydrogel was selected for gastric retention evaluation using the DHSI-IV (NERDT) dynamic gastrointestinal simulation system, as previously described [23,26]. The DHSI-IV system consists of a J-shaped soft silicone stomach, a duodenum module, a programmable peristaltic unit, a digestive fluid delivery system, and a temperature-controlled chamber (Figure 5a). The system was configured to simulate the fasting-state gastric motility and emptying dynamics, with a total experimental duration of 8 h. The simulation cycles were designed to mimic the migrating motor complex (MMC), with Phase I (quiescent phase), Phase II (preliminary activity phase), and Phase III (intestinal clearing phase) timed according to standard human gastric cycles [55]. Specifically, the system was programmed as follows: Phase I for 60 min, Phase II for 30 min, and Phase III for 30 min. This simplification of the natural MMC cycle ensures a standardized, reproducible protocol, as Phase IV (the brief transitional phase) was excluded from the experiments. Phase III was defined as the gastric emptying endpoint. During the simulation, 20 hydrogel particles (with a total volume of 240 mL of water at 37 °C) were introduced into the gastric chamber. Gastric peristalsis and pyloric regulation were simulated by dynamically varying the stomach tilt angle (alternating between 30° and −19.75°, every 30 min) and controlling the pyloric opening diameter, which varied between 8 and 12 mm during the positive tilt angle (30°) and was fixed at 8 mm during the negative tilt angle (−19.75°). These settings replicate the coordinated gastric contractions and motility typically observed under fasting conditions [56]. Although the physiological pyloric diameter in the fed state is typically approximately 2 mm [30], the larger opening was applied to impose a stringent retention challenge. The gastric effluent was collected at hourly intervals, and the remaining hydrogel mass within the stomach chamber was quantified. All experiments were performed at 37 °C in triplicate. Gastric retention was calculated as [56]:

(8) $$\mathrm{Gastric}\;\mathrm{retention}\;\;(\%)\;=\frac{\mathrm{Remaining}\;\mathrm{hydrogel}\;\mathrm{mass}\;\mathrm{at}\;\mathrm{time}\;t}{\mathrm{Initial}\;\mathrm{hydrogel}\;\mathrm{mass}}\times 100\;$$

### 4.6. In Vitro Release Under Simulated Gastric Conditions

Based on its favorable mechanical performance, swelling capacity, and structural integrity under acidic conditions, the CS-P-C 2% hydrogel was selected for in vitro release studies. Release experiments were conducted under simulated gastric conditions to elucidate the influence of network reinforcement, gastric acidity, and peristaltic intensity on diffusion-controlled transport from the hydrogel matrix. The dried hydrogel samples (approximately 0.5 g) were immersed in 60 mL of SGF containing pepsin (4000 U/mL) and maintained at 37 °C to mimic physiological gastric conditions [51]. To simulate different gastric motility intensities, samples were incubated in an orbital shaker at controlled agitation speeds of 75, 100, and 125 rpm, corresponding to low, moderate, and high peristaltic shear, respectively. These agitation levels were chosen to bracket the range of hydrodynamic stresses encountered during fasting-state gastric peristalsis and to evaluate the mechanical robustness of the swollen hydrogel under increasing external shear. The effect of gastric acidity on release behavior was investigated by adjusting the SGF to pH 1.2, 2.2, and 3.2, representing strong acidic conditions during fasting, moderate acidity, and elevated gastric pH typically observed after food intake. This design allowed assessment of acid-responsive network evolution and its impact on diffusion pathways within the hydrogel. At predetermined time intervals (0, 1, 2, 3, 4, 5, 6, and 8 h), 0.1 mL aliquots of the release medium were withdrawn and immediately replaced with an equal volume of fresh SGF to maintain a constant release volume and sink conditions. The concentration of metformin hydrochloride in the collected samples was quantified by UV-visible spectrophotometry at 233 nm. All release experiments were performed at 37 °C in triplicate.

### 4.7. Microstructural and Rheological Analyses During Digestion

Microstructural and viscoelastic evolution of the CS-P-C 2% hydrogels during simulated gastric digestion was investigated under varying acidity (pH 1.2, 2.2, and 3.2) and peristaltic intensities (75, 100, and 125 rpm) to elucidate network adaptation under different gastric environments and mechanical stresses. Hydrogels were collected after 1, 3, 5, and 7 h of incubation to capture time-dependent structural rearrangement associated with swelling, relaxation, and digestion-induced deformation. For microstructural observation, hydrogels were gently removed from the digestion medium, placed on circular aluminum holders, and immediately frozen at −80 °C for 12 h to preserve the swollen network architecture. Samples were subsequently freeze-dried for 24 h (LGD-10D, FTD, Beijing, China). The dried specimens were mounted on aluminum stubs using conductive adhesive and sputter-coated with a thin platinum layer (MC1000, Hitachi Ltd., Tokyo, Japan). Surface and cross-sectional morphologies were examined by scanning electron microscopy (SEM; Hitachi SU1510, Hitachi Ltd., Tokyo, Japan) at an accelerating voltage of 15 kV, enabling visualization of pore structure, network connectivity, and potential structural disruption induced by gastric conditions [57].

The viscoelastic properties of the hydrogels were assessed using a rotational dynamic shear rheometer (Kinexus Pro, Malvern Instruments Ltd., Worcestershire, UK) equipped with a cone-plate geometry (40 mm diameter, CP 4/40) at a fixed gap of 0.2 mm, which was selected to ensure complete sample coverage while minimizing wall slip and edge fracture in the highly swollen hydrogel samples. Frequency sweep measurements were conducted at 37 °C under a low-amplitude shear strain of 1% to ensure linear viscoelastic conditions. The storage modulus (G′) and loss modulus (G″) were recorded over a frequency range of 0.1–100 rad/s, providing quantitative insight into the hydrogel’s elastic (solid-like) and viscous (fluid-like) behavior.

### 4.8. Statistical Analysis

All experiments, including swelling, mechanical, rheological, gastric retention and in vitro release tests, were performed in at least triplicate. Data are expressed as means ± standard deviation (SD). Statistical comparisons among hydrogel samples with different CUD contents were conducted using one-way analysis of variance (ANOVA). When significant differences were detected (*p* ≤ 0.05), Tukey’s post hoc test was applied for multiple comparisons.

## Figures and Tables

**Table 1:** Formulations of core–shell CS/CUR/PAAS hydrogels prepared in this study.

Hydrogel Code	Core Composition			Shell Composition		
	CS (wt.%)	Acetic Acid Solution (0.2 mol/ L, wt.%)	Metformin HCl (wt.%)	PAAS (wt.%)	CUR (wt.%)	NaOH Solution (0.01 mol/ L, wt.%)
CS-P-C 0%	5	94	1	3	0	97
CS-P-C 1%	5	94	1	3	1	96
CS-P-C 2%	5	94	1	3	2	95
CS-P-C 3%	5	94	1	3	3	94
CS-P-C 4%	5	94	1	3	4	93

## Data Availability

Data will be made available upon request.

## Supplementary Materials

The following supporting information can be downloaded at https://www.mdpi.com/article/10.3390/gels12050378/s1, Table S1: Temporal evolution of mean pore radius (μm) under varying experimental conditions; Table S2: Temporal evolution of tan δ (G″/G′) at 1 Hz under varying experimental conditions.
