# Supplementary material for: Curdlan-Reinforced Chitosan/Polyacrylate Interpenetrating Hydrogels with Enhanced Mechanical Stability for Gastric Retention and pH-Responsive Drug Release
Source: Gels. 2026 Apr 30;12(5):378. doi: 10.3390/gels12050378 (PMC13206096; doi:10.3390/gels12050378)
Supplement: Supplementary file 1 [file gels-12-00378-s001.zip › gels-4258215-supplementary.pdf]

Article

Curdlan-Reinforced Chitosan/Polyacrylate Interpenetrating Hydrogels with Enhanced Mechanical Stability for Gastric Retention and pH-Responsive Drug Release

Yuzhong Feng [1], Peng Wu [1,*], Ping Zhang [1], Ni Wang [1], Ke Wang [2], Shuye Qi [3] and Xiaodong Chen [1,4]

[1] Life Quality Engineering Interest Group, School of Chemical and Environmental Engineering, College of Chemistry, Chemical Engineering and Materials Science, Soochow University, Suzhou 215123, China; 20235209003@stu.suda.edu.cn (Y.F.); 20234009042@stu.suda.edu.cn (P.Z.); niwang@suda.edu.cn (N.W.); xdchen@suda.edu.cn (X.C.)

[2] Shenzhen X-Institute, Shenzhen 518055, China; thedogeg@163.com

[3] National Institutes for Food and Drug Control, Beijing 102629, China; qisy@nifdc.org.cn

[4] Institute of Biopharmaceutical and Health Engineering (iBHE), Tsinghua Shenzhen International Graduate School, Tsinghua University, Shenzhen 518055, China

* Correspondence: p.wu@suda.edu.cn

Table S1. Temporal evolution of mean pore radius (μm) under varying experimental conditions

Experimental conditions	1 h	3 h	5 h	7 h
pH 1.2 – 100 rpm	14.94 ± 6.82	20.56 ± 8.98	39.41 ± 17.16	64.91 ± 38.55
pH 2.2 – 100 rpm	17.49 ± 5.30	22.91 ± 7.72	30.80 ± 12.03	34.28 ± 5.89
pH 3.2 – 100 rpm	ND	18.11 ± 7.86	24.18 ± 5.76	25.57 ± 9.18
pH 2.2 – 75 rpm	15.13 ± 4.12	21.05 ± 4.69	24.76 ± 7.94	33.28 ± 11.67
pH 2.2 – 125 rpm	15.07 ± 7.57	19.15 ± 5.97	28.08 ± 6.19	37.11 ± 9.95

ND: Not determined due to the absence of discernible pore structures in SEM images at this time point.

Table S2. Temporal evolution of tan δ (G''/G') at 1 Hz under varying experimental conditions

Experimental conditions	1 h	3 h	5 h	7 h
pH 1.2 – 100 rpm	0.15 ± 0.07	0.09 ± 0.06	0.11 ± 0.09	0.2 ± 0.19
pH 2.2 – 100 rpm	0.33 ± 0.26	0.09 ± 0.05	0.15 ± 0.11	0.14 ± 0.1
pH 3.2 – 100 rpm	0.07 ± 0.03	0.09 ± 0.03	0.11 ± 0.06	0.2 ± 0.12
pH 2.2 – 75 rpm	0.09 ± 0.05	0.11 ± 0.12	0.1 ± 0.11	0.17 ± 0.16
pH 2.2 – 125 rpm	0.19 ± 0.14	0.11 ± 0.09	0.09 ± 0.04	0.09 ± 0.09
